# Stronger together: harnessing natural algal communities as potential probiotics for inhibition of aquaculture pathogens

**DOI:** 10.1128/spectrum.00421-25

**Published:** 2025-05-21

**Authors:** Dóra Smahajcsik, Line Roager, Mikael Lenz Strube, Sheng-Da Zhang, Lone Gram

**Affiliations:** 1 Department of Biotechnology and Biomedicine, Technical University of Denmark 158981 , Lyngby, Denmark; University of Minnesota Twin Cities, St. Paul, Minnesota, USA

**Keywords:** algal microbiome, probiotics, *Vibrio*, *Isochrysis galbana*, *Tetraselmis suecica*, aquaculture, pathogen antagonism

## Abstract

Intensive fish rearing in aquaculture is challenged by infectious diseases, and although vaccines have been successfully developed for mature fish, alternative disease control measures are needed for fish larvae and juveniles. Probiotics offer a promising alternative to antibiotics, with the potential to reduce the risk of antibiotic resistance. Probiotics are typically isolated and used as pure cultures; however, in natural environments, it is the concerted effort of the complex microbiome that keeps pathogens at bay. Here, we developed an *in vitro* assay to evaluate the anti-pathogen efficacy of mixed algal microbiomes from the live feed microalgae *Tetraselmis suecica* and *Isochrysis galbana*. The inhibition of a green fluorescent protein (GFP)-tagged *Vibrio anguillarum*, a key fish pathogen, by microbial communities was measured and quantified as reduction in fluorescence. The *Isochrysis galbana* microbiome was more inhibitory to *V. anguillarum* than the *Tetraselmis suecica* microbiome. During co-culture with the pathogen, the bacterial density of the *Isochrysis* microbiomes increased, while the diversity was reduced as determined by metataxonomic analyses. Bacteria isolated from the fully inhibitory microbiomes were members of *Alteromonadaceae, Halomonadaceae, Rhodobacteraceae, Vibrionaceae, Flavobacteriaceae,* and *Erythrobacteraceae*. Although some strains individually inhibited the pathogen, these were not the key members of the microbiome, and enhanced inhibition was observed when *Sulfitobacter pontiacus* D3 and *Vreelandella alkaliphila* D2 were co-cultured, even though neither was inhibitory as monocultures. Thus, this study demonstrates that microbial communities derived from natural algal microbiomes can have anti-pathogen effects, and that bacterial co-cultures may offer synergistic advantages over monocultures as probiotics, highlighting their promise for aquaculture health strategies.

**IMPORTANCE:**

Aquaculture is the fastest-growing food protein-producing sector, and sustainable disease control measures are required. Probiotics have gained interest as a promising solution for combating fish pathogens, and using mixtures of microorganisms rather than pure cultures may represent a more stable pathogen control. We developed an assay using green fluorescent protein (GFP) tagging of a fish pathogen, enabling the quantitative assessment of the anti-pathogen effects of complex microbiomes. We show that the efficiency of pathogen suppression can be increased with co-cultures compared to monocultures, thus emphasizing the potential in using mixtures of bacteria as probiotics.

## INTRODUCTION

As the human population continues to rise, sustainable production of dietary protein is becoming increasingly important. Aquaculture is a key sector in supplying such high-quality protein and has for decades been the fastest-growing protein-producing food sector (1). However, intensive rearing of fish carries with it the risk of disease outbreaks, which are especially damaging during larval development (1, 2), where disease outbreaks can result in substantial economic losses, as seen in a 1988 incident where vibriosis led to a 40% loss of juvenile turbot in a Norwegian hatchery (3, 4). *Vibrionaceae* infections pose a considerable challenge to marine larviculture and can be caused by a number of *Vibrio* species, including *V. anguillarum*, that can infect up to 50 different fish species (5, 6). Despite advances in vaccine development, vibriosis remains a significant problem in aquaculture, especially due to fish larvae lacking a mature immune system, rendering vaccination ineffective (1, 2).

Many commercially valuable marine fish species require live feed, such as microalgae, at the larval stages since suitable artificial feed formulations are not available (7, 8). However, live feed can serve as the infection vector for pathogenic bacteria in larval rearing, leading to swift and widespread infections and complicating disease management (8, 9). Historically, antibiotics have been used to control these infections among larvae and juveniles, but since this may lead to the spread of antimicrobial resistance (AMR) of both clinical and agri- and aquacultural relevance, alternative approaches are urgently needed (10). A promising strategy to mitigate bacterial pathogen proliferation in live feed and fish larvae is the use of probiotic bacteria which can inhibit pathogenic bacteria (11–13). Probiotic microorganisms, as defined by the Food and Agricultural Organization and the World Health Organization (WHO), are “live microorganisms that, when administered in adequate amounts, confer a health benefit on the host” (14). As reviewed in Sonnenschein et al*.* (15), probiotics can be an environmentally sustainable and economically viable approach to counteract economic losses due to bacterial pathogens in aquaculture and have been intensively studied in the past decades (2, 16). However, the vast majority of such studies have focused on utilizing pure cultures of single probionts (17), which may pose challenges in terms of prolonged establishment of a probiont in live feed microbiomes (18). In contrast to traditional single-isolate probiotics, complex microbiomes may offer a more holistic approach by capitalizing on the synergistic interactions among multiple microorganisms, including bacteria, to effectively combat pathogens (19). Several studies have focused on the use of probiotics in larviculture and have suggested that deriving potential probiotic cultures from the aquaculture system offers an ecological advantage when they are re-introduced (11, 15, 20, 21).

Microalgae, such as *Tetraselmis suecica* and *Isochrysis galbana*, that are used as live feed in many larviculture productions are colonized by a microbiome that can influence the growth and metabolism of the algae (22–24). The algal microbiome depends on the host species (25), is taxonomically diverse, and harbors a large bioactive potential (26, 27). The microbial world is characterized by an intricate web of interactions, some of which are mediated by small molecules through which microorganisms exchange information with their neighbors, competitors, and hosts. These interactions shape the microbial community and its functionality; however, monocultures lack these interactions that are important for, e.g., inducing expression of biosynthetic gene clusters producing bioactive compounds (28–30). We therefore hypothesize that the collective microalgal microbiome and not only pure cultures of probionts could serve to counteract invasion of aquaculture pathogens. Using a complex microbiome to inhibit a target bacterium, *Vibrio anguillarum*, we hope to harvest the enhanced inhibitory properties promoted by the microbiome interactions.

While the study of single culture inhibition is relatively simple, the high-throughput screening of pathogen inhibition by a complex microbiome is more challenging, and a purpose of the present study was to develop a simple screening assay that would allow determining pathogen inhibition by a complex microbiome. Subsequently, we characterized the inhibitory microbiomes by metataxonomic analyses and tested mixtures of bacteria derived from these inhibitory microbiomes for their capability to inhibit the fish pathogen.

## MATERIALS AND METHODS

### Bacterial and algal strains and culture conditions

The fish pathogen *Vibrio anguillarum* strain NB10 with a fluorescent tag integrated into the large chromosome (NB10_pNQFlaC4-gfp27) (31) (later referred to as *Vibrio anguillarum* NB10_gfp) was used as target bacterium in the inhibition assays. The expression of the GFPmut3* gene was controlled by a constitutive promoter P_A1/04/03_ (32). *Phaeobacter piscinae* strain S26 (21) was used as a known antagonist of *Vibrio* as a positive control for establishing the fluorescence-based screening assay. *P. piscinae* and *V. anguillarum* were stored at –80°C and routinely cultivated on Marine Agar (MA; Difco 2216, BD) at 25°C or in Marine Broth (MB; Difco 2216, BD) at 25°C and 200 rpm. MA supplemented with 4 µg/mL chloramphenicol was used to maintain *V. anguillarum* NB10_gfp. All bacterial cultures were stored and cultivated similarly, including *V. anguillarum* NB10 wild type (WT), and bacterial isolates that were obtained from algal cultures and enriched inhibitory microbiomes (see below).

Xenic microalgal cultures of *Tetraselmis suecica* and *Isochrysis galbana* were provided by aquaculture industry partners. Axenic algae *Isochrysis galbana* CCMP 1323 (AxT) and *Tetraselmis suecica* CCAP 66/4 (AxI) were purchased from the Bigelow National Center for Marine Algae and Microbiota (NCMA) and the Culture Collection of Algae and Protozoa (CCAP), respectively, and their axenic status determined by plating on MA. The algae were cultured in f/2 medium without silicate (f/2 – Si; NCMA [33, 34]; made with 3% Instant Ocean [IO; Aquarium Systems Inc., Sarrebourg, France]) and cultivated at 18°C and constant illumination at ~50 μE m^−2^ s^−1^. Cultures were maintained by continuous subculturing every 4 weeks, transferring 1% of the culture to 25 mL fresh medium.

### Fluorescence-based screening assay for GFP-labeled target organism

*V. anguillarum* NB10_gfp was grown overnight as described above, and a 10-fold serial dilution was prepared using MB as a diluent. A total of 180 µL of the dilutions was distributed to the wells of a sterile, black 96-well microplate with flat, clear bottom (Perkin Elmer, 6005430) in technical triplicates. *V. anguillarum* NB10 WT was included as a negative control for the fluorescence measurement, and sterile medium as a no-growth negative control. Fluorescence and absorbance were measured on a BioTek Cytation 5 multi-mode microplate reader at 25°C with shaking (linear, continuous, 567 cpm). Measurements were taken every 20 min over 40 hours, measuring fluorescence (excitation: 485/15 nm, emission: 513/15 nm) and OD_600_.

### Fluorescence-based inhibition assay by *Phaeobacter piscinae* S26

*Phaeobacter piscinae* S26 was co-cultured with *V. anguillarum* NB10_gfp in the fluorescence-based inhibition assay. Overnight cultures of both bacteria were prepared, 10-fold serially diluted in sterile MB, and 90 µL of each dilution of both bacterial cultures was loaded into sterile, black 96-well microplates allowing crossed combinations of the two strains at the different dilutions (total well content of 180 µL). Dilutions of *P. piscinae* S26 were included as a negative control for the fluorescence measurement, and sterile medium as a no-growth negative control. Dilutions of *V. anguillarum* NB10_gfp were included as a positive control for fluorescence. Absorbance and fluorescence of the plates were measured as described above. Bacterial counts of the precultures were determined by 10-fold serial dilution and plating on MA. After the assay, wells showing no fluorescent signal (the inhibitory microbiomes) were 10-fold serial diluted and plated on MA supplemented with 4 µg/mL chloramphenicol to confirm decreased *Vibrio* counts.

### Inhibition of *Vibrio anguillarum* by complex algal microbiomes

**Fig 1:**
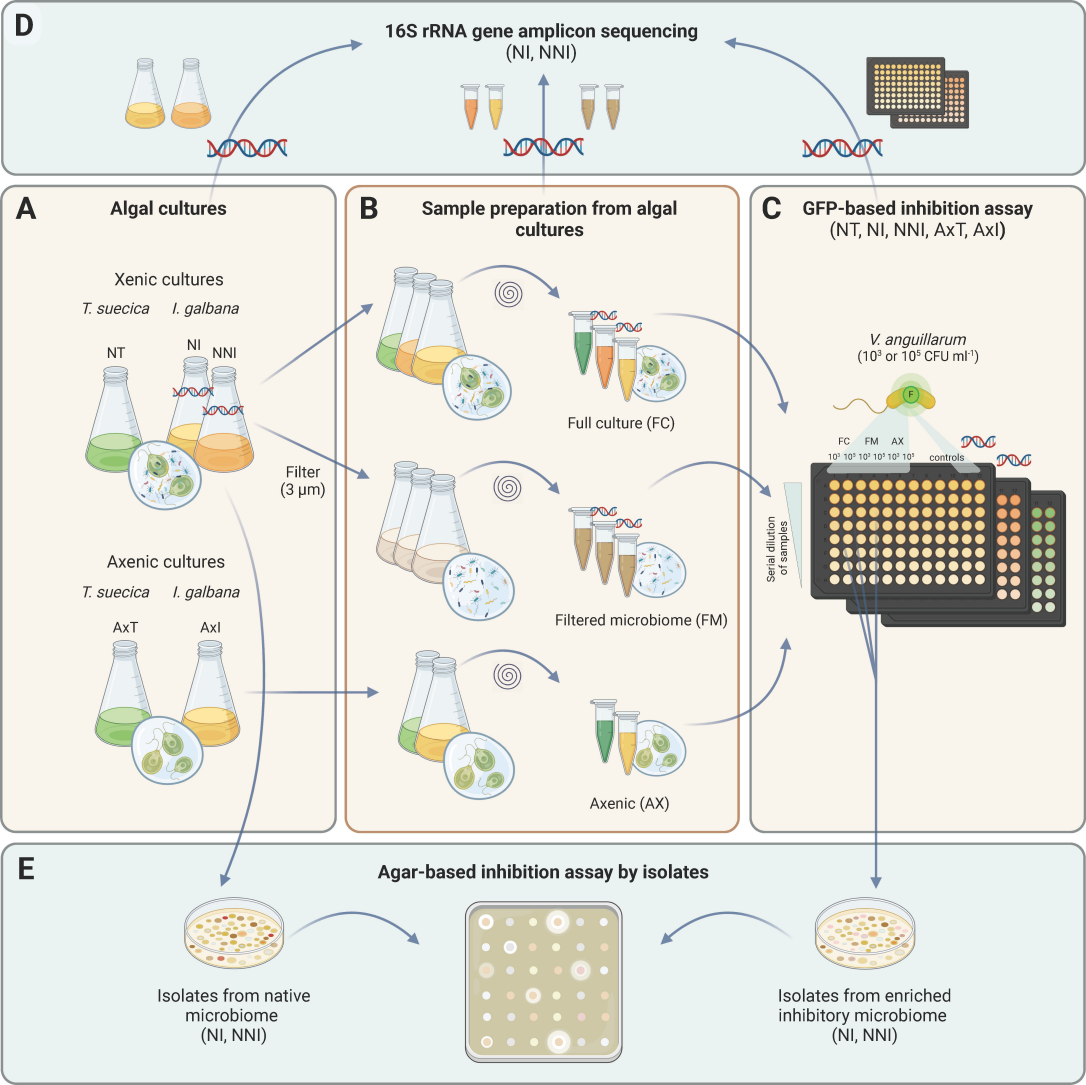
Yellow panels: Overview of algal cultures used in the *Vibrio* inhibition assays. Xenic cultures: *Tetraselmis suecica* (NT) and *Isochrysis galbana* (NI, continuously subcultured; NNI, freshly recruited microbiome). Axenic cultures: *T. suecica* (AxT) and *I. galbana* (AxI) with no microbiome (A). Sample preparation: Xenic cultures were divided into FC (containing algal and bacterial cells) and FM (algal cells removed by filtration) (B). All samples were 100-fold up-concentrated, then 10-fold serial dilutions were co-cultured with *Vibrio anguillarum* NB10_gfp (starting inoculum 103 or 105 CFU mL^-1^) in a GFP-based inhibition assay (C). Teal panels: NI and NNI samples indicated by DNA symbol were analyzed through 16S rRNA gene amplicon sequencing (D). Bacterial isolates from the native microbiome and the enriched inhibitory microbiome of *I. galbana* (NI, NNI) were tested for inhibition of *V. anguillarum* in an agar-based assay (E). (Created using BioRender.)

Microbiomes from two xenic microalgae, *Tetraselmis suecica* and *Isochrysis galbana,* were co-cultured with *V. anguillarum* NB10_gfp using the fluorescence-based screening assay (Fig. 1). Samples from axenic algal cultures were included as controls without microbiomes. Five algal cultures were tested in the assay described above. This included the two axenic cultures (axenic *Tetraselmis* and axenic *Isochrysis*), two xenic cultures of *Tetraselmis suecica* (NT) and *Isochrysis galbana* (NI) that had been re-cultivated in the lab since 2019 and 2020, respectively, and a newly acquired (2023) *Isochrysis galbana* culture (NNI) from an aquaculture collaborator. Cultures (200 mL) of the two microalgae, both axenic and xenic, were prepared by inoculating 10% stock culture to fresh media and incubating them at 18°C and constant illumination for 5 days, reaching approximately 6 log algal cells mL^−1^. The *V. anguillarum* NB10_gfp strain was grown overnight, and algal and bacterial counts were determined at the beginning of the experiment. Bacterial counts (CFU) were determined by 10-fold serial dilution and plating on MA. Plates were incubated for 2 days for *V. anguillarum* and for 4 days for the bacterial counts from algal cultures. All CFU counts were log-transformed before further data handling and plotting. Algal cell counts were determined using microscopy and a Neubauer-improved chamber. To determine if any pathogen inhibition was caused by the algae or by their microbiomes, the xenic algal cultures were divided into two 100 mL fractions: the full culture (FC) with algal and bacterial cells and a filtered microbiome (FM) fraction, where algal cells were removed by filtering (47 mm Ø, pore size 3 µm, mixed cellulose ester, Advantec A300A047A). Before filtering, the cultures were vortexed vigorously to detach bacteria from the algal cells.

To confirm that marine viruses or viral particles did not influence the observed inhibition, the possible pathogen inhibition was tested with an FM and a cell-free supernatant (cf-SN) of NT and NI samples. To prepare the cell-free supernatant, a 100 mL fraction of the FM samples was filtered through 0.22 µm polyethersulfonate filters (Nalgene Rapid-Flow).

To increase the bacterial density of the microbiome (target ~7–8 log CFU mL^−1^), 100 mL of algal cultures was harvested (2,445 × *g*, 10 min, 20°C) and resuspended in 1 mL sterile 3% IO. Ten-fold serial dilutions of both up-concentrated algal fractions (FC and FM; 3% IO as diluent), the cf-SN (3% IO as diluent), and *Vibrio* cultures (MB as diluent) were prepared and loaded into sterile, black 96-well microplates. Combinations of dilutions of the pathogen (target: 3 log CFU mL^−1^ and 5 log CFU mL^−1^) and algal samples (10^−1^ to 10^−5^ dilutions) were loaded. Algal cultures with no addition of the GFP-tagged pathogen were included as a negative control for the fluorescence measurement, and sterile medium as a no-growth negative control. Different dilutions of the pure culture of *V. anguillarum* NB10_gfp were included as a positive control. Fluorescence and OD_600_ were measured as described above. FM samples were measured on a different instrument (BioTek Synergy HTX) than all other samples, resulting in a different scale of the fluorescence measurements. To confirm pathogen inhibition after the assay, wells with fluorescence below 5% of the non-inhibited *Vibrio* control were 10-fold serial diluted and plated on chloramphenicol-supplemented plates (MA). The overview of sample preparation and experimental setup is summarized in Fig. 1.

### Characterizing the enriched inhibitory microbiomes

During the co-culture inhibition assay, the bacterial density of the algal microbiomes was increased. Several dilutions of the algal microbiomes inhibited growth of *V. anguillarum* in a “dose-dependent” manner, and in all co-cultures, the bacterial density of the algal microbiomes increased. These microbiomes which caused suppression of the pathogen will be referred to as enriched inhibitory microbiomes. DNA was extracted from a selection of the wells taking samples from wells exhibiting complete inhibition and wells allowing only marginal growth of *V. anguillarum*. Also, samples were taken from no-growth negative controls of the experiment, resulting in a total of 41 samples. Upon completion of the assay, the plates and samples were stored at −80°C until further processing and then thawed at 4°C, and samples were kept on ice. A total of 2 × 200 µL lysis buffer (400 mM NaCl, 750 mM sucrose, 20 mM EDTA, 50 mM Tris-HCl, 1 mg/mL lysozyme [pH 8.5]) was added to the wells. The contents of the wells were then resuspended and transferred to Eppendorf tubes and incubated at 37°C for 30 min. Proteinase K was added to a final concentration of 100 mg/mL and sodium dodecyl sulfate to a final concentration of 1% vol/vol, and samples were then incubated overnight at 55°C, 300 rpm (35). The order of handling of samples was then randomized before extraction. DNA extraction was done by the Promega Maxwell 16 using the Maxwell LEV Blood DNA Kit, and the quality and purity of the DNA were measured using a DeNovix 439 DS-11+ spectrophotometer (DeNovix Inc., Wilmington, DE, USA) and confirmed by gel electrophoresis.

The V3-V4 region of the 16S rRNA gene of all samples was amplified by PCR as described in Roager et al*.* (36), using the primer set 341f (5'-CCTACGGGNGGCWGCAG-3') and 805r (5'-GACTACHVGGGTATCTAATCC-3') tagged with 30 unique octametric barcodes (37, Table S1). PCR products were purified using Agencourt AMPure XP magnetic beads (0.8:1 bead volume to DNA solution; Agencourt Bioscience Corporation, Beverly, MA, USA). Concentrations of the amplicons were determined using a Qubit 2.0 Fluorometer with the high sensitivity (HS) assay kit (Qubit dsDNA HS assay; Invitrogen by Thermo Fisher Scientific Inc., Eugene, OR, USA). Samples were pooled in equimolar ratios before library preparation and sequencing on an Illumina NovaSeq 6000 platform with paired-end 250 bp reads (Novogene; Cambridge, UK).

### Analyses of sequencing data

Sequencing data analyses were performed using QIIME 2 version 2023.5 (38) and R version 4.2.1 (39). The raw reads were imported to QIIME 2 and demultiplexed, and primers and barcodes were removed using the cutadapt demux-paired plugin (40). Reads were then denoised and dereplicated using the DADA2 package (41), and an amplicon sequence variant (ASV) table was created, leaving out sequences shorter than 400 bp. A phylogenetic tree was constructed using MAFFT (42) and FastTree (43), via the phylogeny plugin. Alpha- and beta-diversity metrics were computed using the diversity plugin (44) at an even sampling depth of 26,226. A classifier trained on the Silva database 138 (45) and the specific primer sequences employed as mentioned above was used for taxonomic classification of the reads via the feature-classifier plugin (46, 47). ASVs which were either not assigned to a phylum, classified as chloroplasts/mitochondria, or had a frequency <10 were filtered out before further analysis in R. Two sterility controls were excluded from the data set before further analysis. QIIME 2 artifacts were imported to R v.4.2.1 using the QIIME 2R package (48), and analysis was continued in RStudio (49) with the tidyverse (50) package. For multivariate analyses using the vegan package, the ASV table was normalized by square-root transformation and Wisconsin standardization (51). Permutational multivariate analyses of variance (PERMANOVA) was then performed using the beta-group-significance function of QIIME 2 and the adonis plugin (52) in R with 999 permutations to test significant differences in beta-diversity as a function of source culture (NI and NNI), degree of pathogen inhibition, microbiome dilution, and sample fraction (FC and FM) based on Bray-Curtis dissimilarities calculated using the normalized ASV table. The analysis was conducted both on the complete data set and on subsets divided by samples before and after the enrichment assay. Interactive effects were included in the models but were removed due to lack of significance.

### Isolation and identification of bacterial strains from *Isochrysis galbana* microbiomes

Culturable bacterial isolates were obtained from a subset of the enriched inhibitory *I. galbana* microbiomes (NI and NNI) analyzed for their microbiome composition. Culture from wells where *V. anguillarum* was inhibited by the microbiomes was serially diluted and plated on MA. Isolates were picked representing colonies with different morphology. Also, strains were picked from the plating of the algal culture before testing inhibition (native microbiome), resulting in a total of 64 bacterial isolates. The strains were re-streaked for purity, and glycerol stocks (20% vol/vol) were made and stored at −80°C. The isolates were later identified by 16S rRNA gene amplification by colony PCR using universal primers 27F and 1492R and subsequent Sanger sequencing by Macrogen (Amsterdam, Netherlands). Sequencing results were compared against the NCBI nucleotide database using the BLASTn function (53) to determine the putative bacterial taxa.

### Inhibition of *V. anguillarum* by pure cultures and co-cultures of bacterial isolates

Antagonism of pure cultures of the 64 bacterial isolates against *V. anguillarum* NB10_gfp was tested using an agar-based assay (54) using 50 µL of culture to 50 mL of agar and spotting 5 µL of an overnight culture of the isolates on the pathogen-embedded plates. Plates were incubated at 25°C and visually inspected for clearing zones after 24 and 48 hours. In a similar manner, dual cultures of isolates from the enriched inhibitory microbiome were spotted on embedded *Vibrio* and inspected for inhibition. Five isolates were chosen for investigating co-culture effects as representatives of the five non-*Vibrio* genera isolated from the enriched inhibitory *I. galbana* microbiomes (Table 2) with complete inhibition of 5.3 log CFU mL^−1^ starting inoculum of *V. anguillarum*. The strains were grown in liquid cultures (MB, 25°C, 200 rpm) for 24 hours and then mixed 1:1 in pairs. A total of 10 µL of the mixtures was spotted on the pathogen-embedded plates. The plates were incubated at 25°C and visually inspected for clearing zones after 24 and 48 hours. Monocultures of the individual strains mixed 1:1 with MB were used as controls. To validate the results by the co-culture of isolates D2 and D3, their inhibition was tested against a selection of nine strains of *Vibrio anguillarum* (90-11-286, DSM21597, PF7, PF4, 9014/8, S2 2/9, 2NB10 and 775)4299, with different virulence against fish larvae (55). Monocultures and co-cultures were spotted on the pathogen-embedded plates in biological triplicates, as described above. Biological triplicates of isolate H2 (*Phaeobacter piscinae*) were used as a positive control.

### Whole-genome sequencing, annotation, and analysis

The five isolates selected for the co-culture study were whole-genome sequenced with Oxford Nanopore sequencing, using the GridION platform. Genomic DNA was extracted from overnight cultures with the NucleoSpin Tissue Mini kit (Macherey-Nagel, 740952.5) protocol for bacteria, with 3 hours of incubation during pre-lysis. The quality, purity, and concentration of the DNA were assessed as described for amplicon sequencing. The library was prepared using the Rapid Barcoding Kit 24 V14 (SQK-RBK114.24). The resulting reads were processed by the Dragonflye pipeline (56). Briefly, adaptors were trimmed using Porechop, and reads smaller than 1,000 bp were removed with Nanoq. The genomes were then assembled with Flye and polished with Medaka. The assemblies were then annotated with Bakta v.1.8.2 (57) and profiled for BGCs with antiSMASH 7.0 (58). Subsequently, the assembled genomes were taxonomically classified using autoMLST (59) and average nucleotide identity (ANI).

## RESULTS

### Fluorescence-based inhibition assay for GFP-labeled *Vibrio anguillarum*

**Fig 2:**
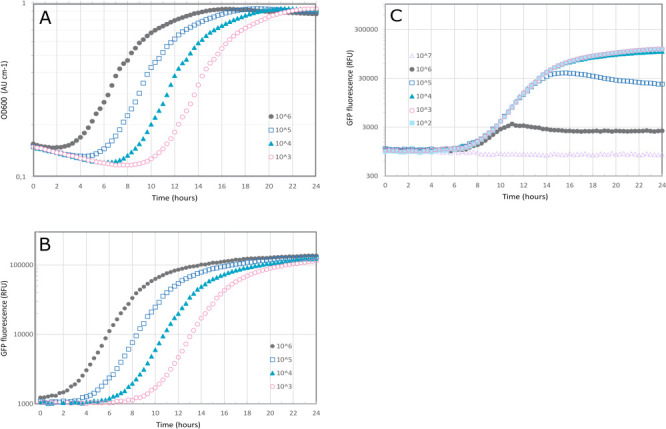
*Vibrio anguillarum* NB10_gfp at different initial concentrations in CFU mL^-1^, as measured by (A) absorbance at 600 nm and by (B) fluorescence (GFP) using excitation of 485/15 nm and emission of 513/15 nm, and the (C) inhibition profile of *V. anguillarum* NB10_gfp (starting inoculum: 10^4^ CFU mL^-1^) by antagonist *Phaeobacter piscinae* S26 at different initial concentrations in CFU mL^-1^, as measured by fluorescence (GFP, excitation: 485/15 nm, emission: 513/15 nm).

*V. anguillarum* NB10_gfp, as expected, grew well in MB, reaching an absorbance of 0.929 ± 0.003 at 600 nm (in 19 hours for 5.3 log CFU mL^−1^ starting inoculum). The starting inocula of *V. anguillarum* NB10_gfp ranged between 1.3 and 9.3 log CFU mL^−1^. In pure culture, the growth of *V. anguillarum* could be followed by the fluorescent signal indicating the same lag phase, growth rate, and stationary phase entry as seen on the OD_600_ measurements (Fig. 2A and B). *V. anguillarum* was then co-cultured with a dilution series of a known antagonist, *Phaeobacter piscinae* S26, in biological triplicates, to provide proof of concept that growth inhibition of the pathogen could be detected by measuring (lack of) fluorescence signal of a fluorescently tagged target organism. The starting inocula of *P. piscinae* S26 ranged between 9.1 and 1.1 log CFU mL^−1^. As expected, lower starting concentrations of *Vibrio* were more easily inhibited than higher concentrations (data not shown), and, conversely, higher concentrations of *Phaeobacter* were more inhibitory than lower concentrations (Fig. 2C). Quantifying *Vibrio* on chloramphenicol-supplemented plates after the assay, from the wells with fluorescent signal <5% of the *Vibrio* positive control, confirmed inhibition of the pathogen (<4.34 ± 0.63 log CFU/mL).

### Anti-pathogen inhibitory effect of algal microbiomes on *Vibrio anguillarum*

To investigate the inhibitory effect of their microbiomes on *V. anguillarum* NB10, dilutions of samples from cultures of two xenic microalgae, *I. galbana* and *T. suecica,* were co-cultured with the GFP-tagged fish pathogen. In the experiments, the starting inocula of *V. anguillarum* NB10_gfp was 3.1 ± 0.3 log CFU mL^−1^ (target: 3 log CFU mL^−1^) and 5.1 ± 0.3 log CFU mL^−1^ (target: 5 log CFU mL^−1^). Initial algal cell counts varied between 5.2 and 6.4 log cells mL^−1^, and up-concentration resulted in approximately 1.5 log increase to between 7.2 and 8.0 log cells mL^−1^. Initial bacterial counts in the *Isochrysis* samples varied between 5.7 and 6.6 log CFU mL^−1^, in the up-concentrated FC fraction between 7.6 and 8.4 log CFU mL^−1^, and in the up-concentrated FM fraction between 7.6 and 8.1 log CFU mL^−1^ (Table S2).

**Fig 3:**
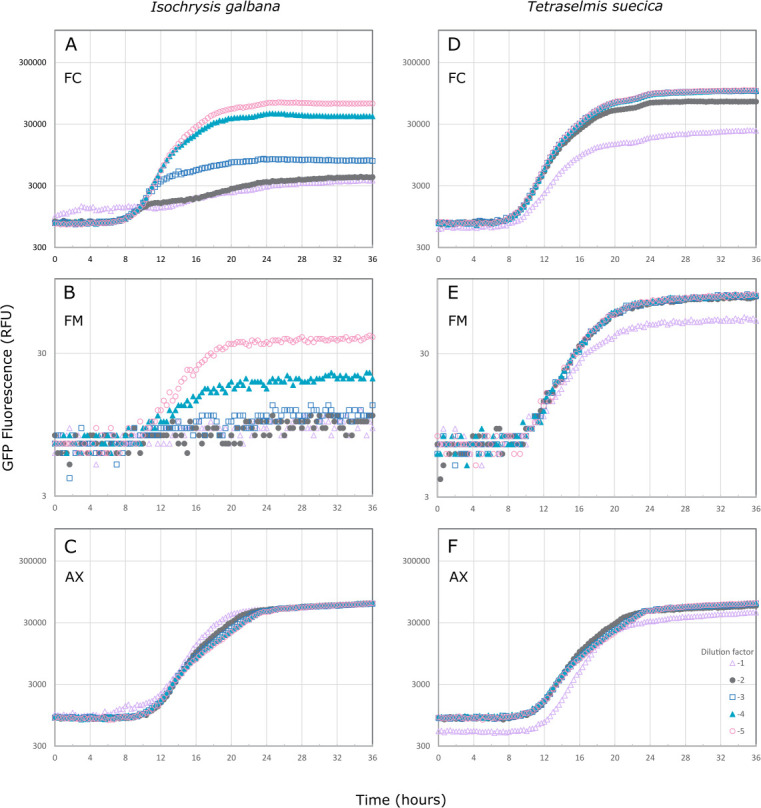
Inhibition by *Isochrysis galbana* (left) and *Tetraselmis suecica* (right) microbiomes of *Vibrio anguillarum* NB10_gfp (starting inoculum of 3.1 ± 0.3 log CFU mL^-1^), as measured by fluorescence (GFP, excitation: 485/15 nm, emission: 513/15 nm). The inhibitory effect of serial dilutions of different fractions of the algal cultures has been tested: FC (3A and 3D), with algal and bacterial cells; FM (3B and 3E), where algal cells have been removed; and axenic (AX; 3C and 3F) cultures, where the algae cells are free of bacteria. FM samples were measured on a different instrument, hence a different scale of relative fluorescence unit, RFU.

Microbiomes from both *T. suecica* and *I. galbana* inhibited growth of *V. anguillarum*, with the *I. galbana* microbiome being more potent (Fig. 3A vs 3D and 3B vs 3E). The same inhibition pattern was observed for the filtered microbiomes (algal cells removed) as for the full culture (algal and bacterial cells) (Fig. 3A vs 3B and 3D vs 3E). No inhibition was observed from samples from axenic algae cultures (Fig. 3C and 3F) or cell-free supernatants (data not shown), indicating that it is indeed the microbiomes growing in the wells that inhibit the pathogen. Plating on chloramphenicol-supplemented plates from wells with decreased fluorescent signal confirmed that if the GFP signal was less than 10% of the control, the *Vibrio* was inhibited, with an abundance of <5.67 ± .75 log CFU/mL, as opposed to approximately 9 log CFU/mL in the non-inhibited control. OD_600_ measurements can be seen in Fig. S1.

### Sequence-based analysis of microbiomes

In total, 70,542,064 paired-end reads were obtained from the 16S rRNA gene amplicons, with a mean of 1,500,894 reads per sample. After denoising, merging, filtering out chloroplasts and mitochondria, removal of short sequences (<400 bp), and low abundant ASVs (total frequency <10), this resulted in a total of 320 ASVs, with a mean read count per sample of 209,507 (Table S3).

### Microbiome composition and diversity analyses

**Fig 4:**
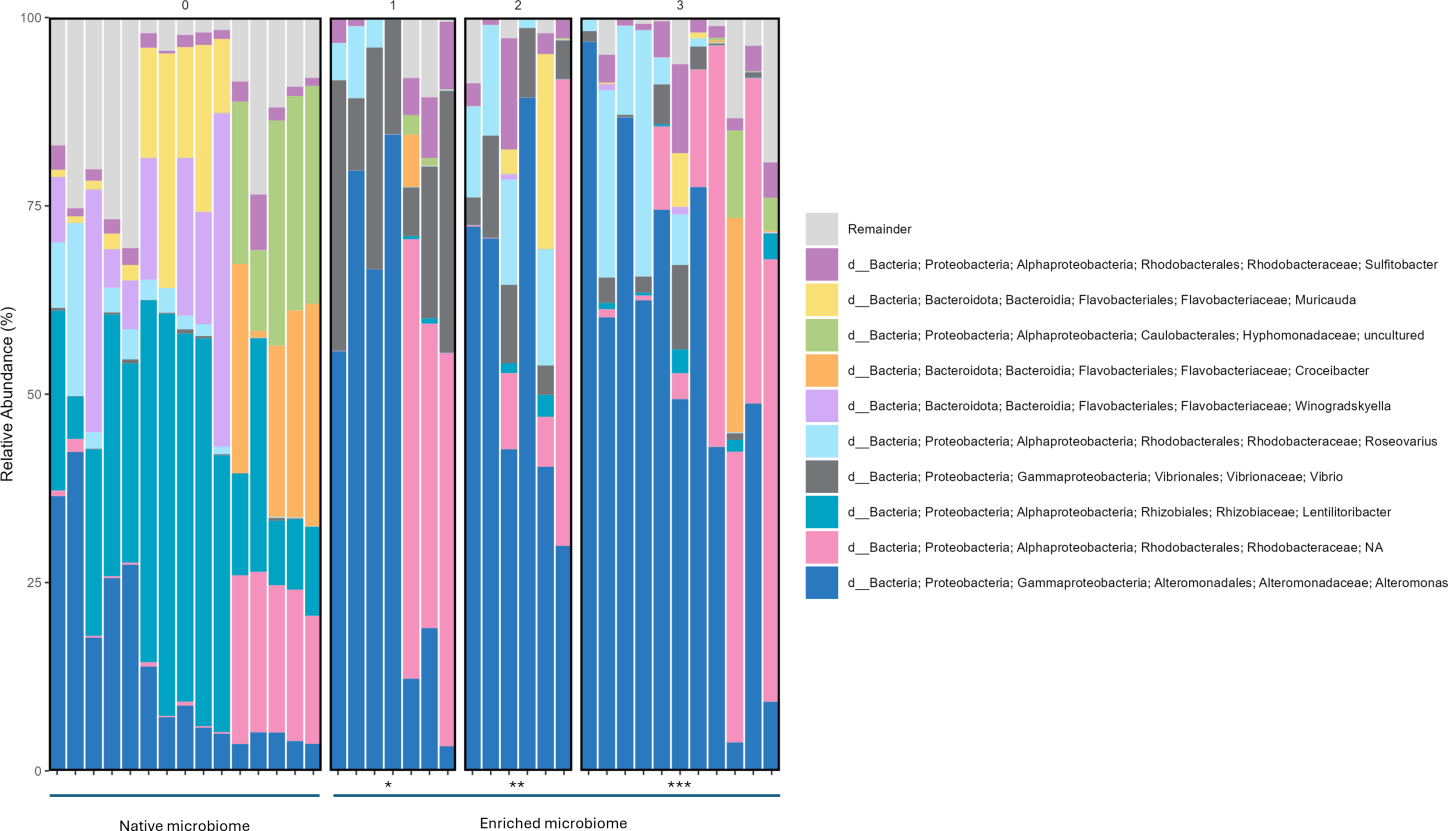
Microbial composition (top 10 most abundant genera) of the native (left) and enriched inhibitory (right) microbiomes based on the 16S rRNA amplicon sequencing results. Samples from the enriched inhibitory microbiome are grouped by their inhibition profile: marginal growth of pathogen (*), medium inhibition of pathogen (**), full inhibition of pathogen (***).

The native microbiomes of *I. galbana* were dominated by *Alteromonadaceae, Rhizobiaceae, Flavobacteriaceae, Rhodobacteraceae,* and *Hyphomonadaceae* (Fig. 4). The microbiome composition of the two *I. galbana* cultures (NI and NNI) differed significantly (PERMANOVA, *R*^2^ = 0.312, *P* = 0.001), with NNI representing a relatively freshly recruited microbiome and NI representing a semi-adapted microbiome, subcultured many times in the laboratory over 4 years. The two cultures shared several genera, such as *Flavobacteriaceae, Rhizobiaceae, Alteromonadaceae,* and *Rhodobacteraceae*, but also had distinct microbiome compositions. *Hyphomonadaceae* and *Cyclobacteriaceae* were unique to NI, and each culture had several distinct representatives of *Flavobacteriaceae* and *Rhodobacteraceae* (Fig. S1 ; Fig. 4).

The enriched inhibitory microbiomes sampled after the *Vibrio* inhibition assay were largely dominated by *Alteromonadaceae* and *Rhodobacteraceae*, with a higher relative abundance of *Vibrionaceae* compared to the native microbiome (Fig. 4). As expected, the relative abundance of *Vibrionaceae* was highest in samples with marginal growth of the pathogen, compared to those with complete inhibition of the target pathogenic vibrio. However, *Vibrionaceae* were also detected in lower relative abundances in enriched cultures that fully inhibited *V. anguillarum*, suggesting these sequences could represent the growth of native vibrios of the algal microbiome. The relative abundances of *Alteromonadaceae* and *Rhodobacteraceae* increased during the inhibition assay, whereas those of *Rhizobiaceae*, *Flavobacteriaceae*, and *Hyphomonadaceae* decreased.

Community richness (Faith index), diversity (Shannon index), and evenness (Pielou’s index) showed no significant differences between microbiomes with different degrees of pathogen inhibition (*P* > 0.05). However, based on a pairwise Kruskal-Wallis analysis, significant differences in Pielou’s index were observed between the NI and NNI cultures (*P* = 0.0041), with the NI culture having a higher diversity and evenness. PERMANOVA analysis of the full data set indicated a significant shift in microbial community composition from before (native microbiome) to after the experiment (enriched inhibitory microbiome). Therefore, the data were split into two subsets for further analysis.

Post-enrichment, community composition was significantly affected by the source culture (NI vs NNI) (PERMANOVA, *R*^2^ = 0.253, *P* = 0.001), degree of pathogen inhibition (PERMANOVA, *R*^2^ = 0.064, *P* = 0.017), microbiome dilution (PERMANOVA, *R*^2^ = 0.098, *P* = 0.001), and algal fraction (FC vs FM) (PERMANOVA, *R*^2^ = 0.060, *P* = 0.025). Residual variation accounted for 52.5% of the total variation.

In conclusion, the degree of pathogen inhibition was associated with differences in the taxonomic composition of the sampled *Isochrysis* microbiomes. However, most of the variation between microbiomes was explained by differences between the two source cultures (NI and NNI).

### Isolation and identification of bacterial strains from *Isochrysis galbana* microbiomes

A total of 64 isolates were obtained from *I. galbana* microbiomes either from the native algal culture (19 strains) or from the enriched inhibitory microbiomes after *Vibrio* inhibition (45 strains). These were tentatively identified using full-length 16S rRNA gene sequencing (Table S4). Strains from the native microbiome were identified as members of the *Alteromonas* (two isolates)*, Croceibacter* (one isolate)*, Mameliella* (one isolate)*, Phaeobacter* (three isolates)*, Qipengyuania* (three isolates)*, Roseovarius* (two isolates)*, Ruegeria* (one isolate), and *Sulfitobacter* (six isolates) genera (Table 1), whereas strains from the enriched inhibitory microbiomes were identified as members of the *Alteromonas* (5 isolates)*, Halomonas* (1 isolate)*, Phaeobacter* (18 isolates)*, Roseovarius* (7 isolates)*, Sulfitobacter* (11 isolates), and *Vibrio* (3 isolates) genera (Table 2). The *Halomonas* isolate was later identified using whole-genome sequencing and ANI as *Vreelandella alkaliphila* (previously *Halomonas alkaliphila*).

**TABLE 1:** Identification (16S rRNA gene sequence and BLAST) of bacterial isolates from the native *Isochrysis galbana* cultures

Top BLAST hit (NCBI)	Genus	Family	Number of isolates	Present in enrichment culture
*Alteromonas* sp.	*Alteromonas*	*Alteromonadaceae*	2	Yes
*Croceibacter* sp.	*Croceibacter*	*Flavobacteriaceae*	1	No
*Mameliella* sp.	*Mameliella*	*Rhodobacteraceae*	1	No
*Phaeobacter* sp.	*Phaeobacter*	*Rhodobacteraceae*	3	Yes
*Qipengyuania* sp.	*Qipengyuania*	*Erythrobacteraceae*	3	No
*Roseovarius nubinhibens*	*Roseovarius*	*Rhodobacteraceae*	2	Yes
*Ruegeria* sp.	*Ruegeria*	*Rhodobacteraceae*	1	No
*Sulfitobacter* sp.	*Sulfitobacter*	*Rhodobacteraceae*	6	Yes

**TABLE 2:** Identification (16S rRNA gene sequence and BLAST) of bacterial isolates from the enriched inhibitory *Isochrysis galbana* cultures after co-culture with *Vibrio anguillarum* NB10_gfp

Top BLAST hit (NCBI)	Genus	Family	Number of isolates	In full (**) or marginally (*) inhibitory enrichments	
				**	*
*Alteromonas* sp.	*Alteromonas*	*Alteromonadaceae*	5	Yes	No
*Halomonas alkaliphila*	*Halomonas*	*Halomonadaceae*	1	Yes	No
*Phaeobacter* sp.	*Phaeobacter*	*Rhodobacteraceae*	18	Yes	Yes
*Roseovarius nubinhibens*	*Roseovarius*	*Rhodobacteraceae*	7	Yes	Yes
*Sulfitobacter* sp.	*Sulfitobacter*	*Rhodobacteraceae*	11	Yes	Yes
*Vibrio* sp.	*Vibrio*	*Vibrionaceae*	3	Yes	Yes

### Inhibition of *Vibrio anguillarum* by pure cultures and co-cultures of bacterial isolates

**Fig 5:**
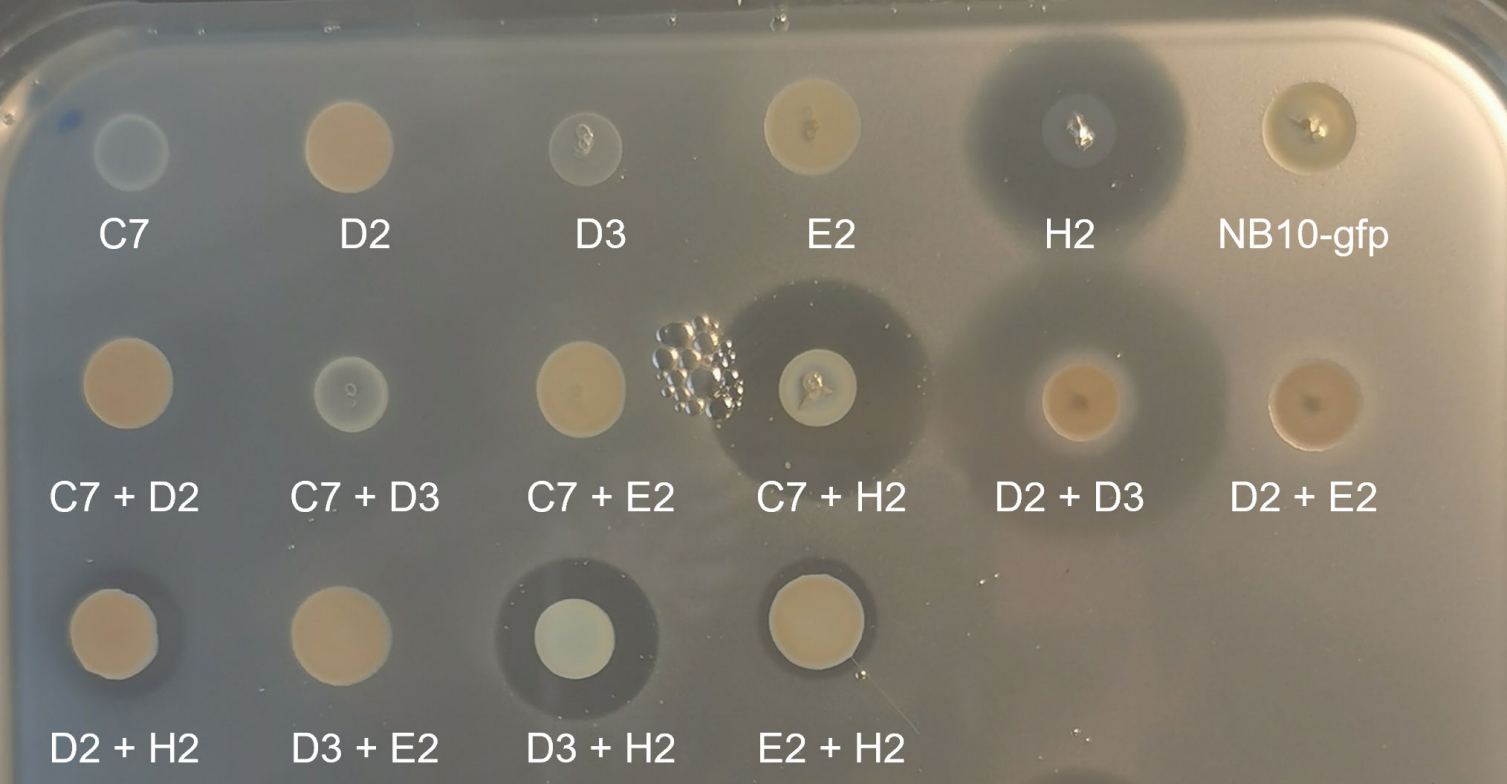
Inhibition of *Vibrio anguillarum* NB10_gfp by co-cultures of bacterial species isolated from the enriched inhibitory *Isochrysis galbana* cultures, in an agar-based pathogen embedding assay. C7, *Roseovarius* sp.; D2, *Vreelandella alkaliphila*; D3, *Sulfitobacter pontiacus*; E2, *Alteromonas macleodii*; H2, *Phaeobacter piscinae*.

Pure cultures of all 64 bacterial isolates were tested for their ability to inhibit *V. anguillarum* NB10, and 17 isolates with pronounced inhibitory activity (zone of inhibition in the embedded pathogen) were all identified as *Phaeobacter* sp. (Table 3, Fig. S3). The dual co-cultures of representative isolates showed inhibition zones in all co-cultures that included *Phaeobacter piscinae* (isolate H2), although the inhibition zone in co-cultures with some strains (isolate E2) was reduced. Of special interest was the co-culture of *Sulfitobacter pontiacus* (isolate D3) and *Vreelandella alkaliphila* (isolate D2), which, despite showing only slight (*Vreelandella alkaliphila*) or no (*Sulfitobacter pontiacus*) inhibition in single culture, were remarkably inhibitory when grown together (Fig. 5). Monocultures and co-cultures of these two isolates were subsequently tested against eight additional *V. anguillarum* strains with different virulence, and the co-culture showed varying degrees of inhibition against *V. anguillarum* 775, 4299, and PF7 (Table S5). Isolate H2 showed strong inhibition against all *V. anguillarum* strains.

**TABLE 3:** Bacterial strains isolated from *Isochrysis galbana* cultures with the ability to inhibit *V. anguillarum*^*a*^

Isolate	Source	Best BLAST hit	ID%	Inhibition of *Vibrio anguillarum* NB10
B7	Raw culture, NI	*Phaeobacter piscinae*	99.57%	++
B9	Raw culture, NI	*Phaeobacter piscinae*	99.43%	+
C3	FC, NNI	*Phaeobacter piscinae*	99.43%	+
D4	FC, NNI	*Phaeobacter piscinae*	99.14%	+
D6	FC, NI	*Phaeobacter piscinae*	99.24%	+++
E4	FC, NI	*Phaeobacter piscinae*	99.42%	+++
E6	FC, NI	*Phaeobacter sp*. M8-4.3	98.24%	+++
E8	FC, NI	*Phaeobacter piscinae*	99.42%	++
E9	FC, NI	*Phaeobacter piscinae*	98.90%	+++
F3	FC, NI	*Phaeobacter sp*. M8-4.3	98.07%	++
F6	FM, NNI	*Phaeobacter piscinae*	98.72%	+
H2	FM, NI	*Phaeobacter piscinae*	98.96%	+++
H3	FM, NI	*Phaeobacter piscinae*	99.33%	*
H8	FM, NI	*Phaeobacter piscinae*	99.33%	++
I1	FM, NI	*Phaeobacter piscinae*	99.14%	+
I2	FM, NI	*Phaeobacter piscinae*	99.04%	++
I3	FM, NI	*Phaeobacter piscinae*	99.24%	+++

^*a*^ All isolates characterized by a clearing zone of different sizes (+, ++, +++) or a faint clearing zone (*) were identified as *Phaeobacter* sp.

### Whole-genome sequencing and genome mining

The species identity of the five chosen isolates was confirmed through ANI% analysis: *Roseovarius nubinhibens* (isolate C7, ANI 90.0%; referred to as *Roseovarius* sp.), *Vreelandella alkaliphila* (isolate D2, ANI 98%), *Sulfitobacter pontiacus* (isolate D3, ANI 97.3%), *Alteromonas macleodii* (isolate E2, ANI 99.0%), and *Phaeobacter piscinae* (isolate H2, ANI 98.2%). Genome assembly of the sequencing data from the five isolates yielded three closed genomes and two draft genomes (Table 4). According to antiSMASH (58) analysis, *Vreelandella alkaliphila* D2 carries seven BGCs (NI-siderophore, ranthipeptide, redox-cofactor, RIPP-like, T1PKS, betalactone, ectoine) and *Sulfitobacter pontiacus* D3 four BGCs (RiPP-like, betalactone, hserlactone, redox-cofactor), potentially encoding antibacterial compounds (Table S6). The structure of compounds cannot be accurately predicted due to the low similarity compared to the known BGCs in MIBiG (Table S6).

**TABLE 4:** Whole-genome sequencing results of five bacterial isolates from the *Isochrysis galbana* microbiome^*a*^

Isolate	Total genome length (bp)	N50 (bp)	Number of contigs	GC content (%)	Status of genome
*Roseovarius* sp. C7	3,819,388	3,578,952	6	62.9	Closed
*Vreelandella alkaliphila* D2	4,408,683	4,359,254	3	52.8	Closed
*Alteromonas macleodii* E2	5,090,176	4,653,070	3	44.5	Closed
*Sulfitobacter pontiacus* D3	3,995,328	279,876	45	60.2	Draft
*Phaeobacter piscinae* H2	3,981,236	909,707	11	60.2	Draft

^*a*^ Whole-genome sequencing of *Roseovarius* sp. C7, *Vreelandella alkaliphila* D2, *Sulfitobacter pontiacus* D3, *Alteromonas macleodii* E2, and *Phaeobacter piscinae* H2 yielded three closed genomes and two draft genomes.

## DISCUSSION

The rapid spread of pathogenic bacteria within intensive aquaculture systems remains a significant challenge, especially during the vulnerable larval development stage, and disease outbreaks often result in substantial economic losses. In this study, we investigated the probiotic potential of the microbiomes from two algae typically employed as live feed in marine larviculture, *Tetraselmis suecica* and *Isochrysis galbana*. By identifying microbiomes with disease suppression, we aim to provide a novel approach to disease control in aquaculture, reducing reliance on antibiotics and the risk of the spread of AMR.

Determining the inhibitory effect of a mixed microbiome against a particular pathogenic bacterium requires specific detection and quantification of the pathogen. Previous works on fish pathogens have used several approaches for determining inhibition in a mixed microbial population. For instance, tagging the pathogen with an antibiotic resistance marker will enable specific quantification on antibiotic-containing media (11, 20, 31), or the pathogen can be counted on semi-selective media where it appears with distinct colony morphology (8, 60, 61). Other approaches have used flow cytometry to determine concentrations of a GFP-tagged pathogen (20), or have developed specific primers and a qPCR approach for specific detection (8). Both the culturing approaches as well as the qPCR method are labor-intensive and time-consuming. To enable a faster screen for pathogen inhibitory effect of a mixed microbial community, we used a GFP-tagged pathogen and demonstrated that the fluorescent signal is a robust indication of growth of the pathogen, and that it can be used to detect its inhibition by other microorganisms. Thus, we developed a simple methodology for screening mixed microbiomes for their anti-pathogenic potential, thereby offering a valuable tool for developing sustainable disease management strategies also outside of the aquaculture sector. The method facilitates the identification and selection of microbiomes with the greatest potential for disease control, contributing to the sustainable growth of aquaculture and the global effort to combat AMR.

We used the method to demonstrate that both the native, complete microbiomes and diluted microbiomes of two microalgae used in aquacultures were able to inhibit the growth of the fish pathogen *Vibrio anguillarum*. In contrast to findings from previous studies where cultures of *Isochrysis* sp. and *Tetraselmis suecica* have themselves been inhibitory to fish pathogenic *Vibrio* species (60–62), we did not find that *V. anguillarum* was inhibited by axenic *I. galbana* or *T. suecica,* nor by the cell-free supernatants of either culture. Thus, in our study, it was clearly the microorganisms associated with the algae that inhibited the growth of the pathogen, emphasizing the crucial role of microbial communities associated with live feed in larviculture for disease control. These findings highlight the potential of harnessing natural microbial consortia to enhance disease resistance in aquaculture settings. Furthermore, in our experiments, the *Isochrysis* microbiome inhibited the growth of the pathogen (relative fluorescence unit, RFU < 10% of control) at 1:10 (*Vibrio:Isochrysis* microbiome) starting inoculation ratio, showing a more pronounced inhibitory potential as compared to that of a monoculture of *P. piscinae* S26, where it took an inoculation ratio of 1:100 (*Vibrio:P. piscinae* S26) to reach a similar reduction of the RFU, supporting our hypothesis that a mixed microbiome may be more inhibitory to a pathogen than individual pure cultures.

Sequence-based analysis of *I. galbana* microbiomes found that *Rhizobiaceae*, *Alteromonadaceae*, and *Flavobacteriaceae* family members dominated the native *I. galbana* microbiome. This is in line with a number of previous studies reporting these families as members often found in the core microbiome of microalgae (36, 63, 64). The native microbiome also harbored members of the *Rhodobacteraceae*, which is a family commonly associated with microalgae and algal blooms (36, 63–66). The enriched inhibitory microbiomes were dominated by *Alteromonadaceae*, *Rhodobacteraceae*, and *Vibrionaceae* members, with decreased relative abundances of *Rhizobiaceae* and *Flavobacteriaceae* compared to the native microbiome. These shifts are likely heavily influenced by transferring the algal microbiome to a nutrient-rich assay medium (MB) favoring fast-growing, heterotrophic bacteria. Interestingly, the degree of pathogen inhibition by the enriched inhibitory microbiomes is not explained by differences in the community richness, diversity, and evenness. However, it does correlate with distinct patterns in the overall community composition, suggesting that the inhibitory potential is influenced by the structure of the microbiome and synergistic interactions within a diverse community. Our findings also imply that complete pathogen inhibition can be achieved by a range of different enrichment cultures.

Pure cultures of *Phaeobacter piscinae* H2 isolated from the *I. galbana* microbiome inhibited *V. anguillarum* when tested in an agar-based assay, and this is consistent with numerous previous studies showing that several *Phaeobacter* species can inhibit *Vibrio* species (20, 21, 67–69). Notably, all isolates that produced an inhibition zone of the embedded *V. anguillarum* were identified as *Phaeobacter* species; however, this genus was not among the 10 most abundant genera of the enriched inhibitory microbiomes. This could indicate that instead of the potent antagonistic effect of particular genera, it may be a synergistic effect of the microbiome members that inhibits the pathogen. The *Phaeobacter* inhibition was reduced when co-cultured with *Alteromonas macleodii* isolate E2. The reason for this reduction is not known but could be caused by a reduction in the production of the *Phaeobacter* antibacterial compound, tropodithietic acid (TDA) , as recently observed when a TDA-producing *Phaeobacter* was co-cultured with a *Pseudoalteromonas piscicida* (70).

The co-culture of *Sulfitobacter pontiacus* D3 and *Vreelandella alkaliphila* D2, isolated from the *I. galbana* microbiome, was inhibitory to *V. anguillarum* NB10, despite neither isolate showing pathogen inhibition as pure cultures. The co-culture also showed varying degrees of inhibition against three additional *V. anguillarum* strains, each of which, importantly, were phylogenetically distinct according to autoMLST analysis . This indicates that the observed inhibition is not limited to low-virulence strains, but also that the effect may not be universal and that some pathogens are not affected. While it is beyond the scope of the current study to unravel the molecular and chemical mechanisms underlying this co-culture-induced inhibition, previous research has demonstrated that BGCs encoding antibiotic compounds can be elicited by co-cultivation of bacteria (28–30, 71). According to antiSMASH analyses, the genomes of both bacteria harbor several BGCs. Therefore, future metabolomic analyses of the mono- and co-cultures could provide insight into the co-culture-induced antimicrobial mechanism. Moreover, several *Halomonas* species have been reported to produce an array of hydrolytic enzymes, extracellular polysaccharides (EPS) (72), antibiotics (73), antifungals (74), and pigments (75). *Vreelandella alkaliphila* D2, in particular, carries seven biosynthetic gene clusters potentially encoding antibacterial compounds. This, combined with the lack of inhibition by the monoculture, could suggest that the presence of *Sulfitobacter pontiacus* D3 may trigger the inhibitory effect. *Sulfitobacter* species, often associated with marine algae and corals (76–78), possess the ability to metabolize sulfite (79–81) and dimethylsulfoniopropionate (DMSP) (76). The breakdown of DMSP produces potentially antimicrobial compounds such as acrylate (82, 83) and dimethyl sulfide, which could indicate that the sulfur metabolism capabilities of *Sulfitobacter* may contribute to pathogen inhibition. Several *Sulfitobacter* species have also been reported to inhibit fungal pathogens (84) and produce EPS (85) and extracellular cyclodipeptides (86).

Beiralas et al. reported that *Sulfitobacter pontiacus* is a key species of the *Emiliania huxleyi* microbiome, protecting its algal host from bacterial pathogens even at low initial abundance (78). While the mechanism of the algal protector phenotype was not determined, the authors suggest that harboring *Sulfitobacter* species in the algal microbiome presents the host with an advantage and that *Sulfitobacter* species may play a key role in influencing population dynamics. Building on these findings, we speculate that even a low abundance of *Sulfitobacter pontiacus* in a community or co-culture may induce synergistic effects that enhance pathogen inhibition, potentially mediated through the production of bioactive compounds. These insights highlight the importance of microbial interactions in community functionality and stability.

In conclusion, by introducing an efficient methodology for screening mixed microbiomes, our study offers a promising approach for identifying candidate microbiomes for sustainable disease management. Our findings highlight the potential of harnessing natural microbial consortia to enhance disease resistance in aquaculture and support the importance of microbial co-cultures as potential disease control strategies.

## Data Availability

The sequencing data from the microbiome analysis and the assembled whole genomes are available in the NCBI Sequence Read Archive under BioProject ID PRJNA1150956.

## SUPPLEMENTAL MATERIAL

Supplemental tables and figures spectrum.00421-25-s0001.docx Tables S1 to S5; Figures S1 to S3.

ASM does not own the copyrights to Supplemental Material that may be linked to, or accessed through, an article. The authors have granted ASM a non-exclusive, world-wide license to publish the Supplemental Material files. Please contact the corresponding author directly for reuse.
